# Genetic gains with rapid‐cycle genomic selection for combined drought and waterlogging tolerance in tropical maize (*Zea may*s L.)

**DOI:** 10.1002/tpg2.20035

**Published:** 2020-07-20

**Authors:** Reshmi R. Das, M. T. Vinayan, Manish B. Patel, Ramesh K. Phagna, S. B. Singh, J. P. Shahi, Akashi Sarma, N. S. Barua, Raman Babu, K. Seetharam, Juan A. Burgueño, P. H. Zaidi

**Affiliations:** ^1^ CIMMYT Asia Maize Program ICRISAT Campus Hyderabad 502324 India; ^2^ Anand Agricultural University Godhara India; ^3^ ICAR Indian Institute of Maize Research Ludhiana India; ^4^ Banaras Hindu University Varanasi India; ^5^ Assam Agricultural University Jorhat India; ^6^ CIMMYT El‐Batan Mexico

## Abstract

Rapid cycle genomic selection (RC‐GS) helps to shorten the breeding cycle and reduce the costs of phenotyping, thereby increasing genetic gains in terms of both cost and time. We implemented RC‐GS on two multi‐parent yellow synthetic (MYS) populations constituted by intermating ten elite lines involved in each population, including four each of drought and waterlogging tolerant donors and two commercial lines, with proven commercial value. Cycle 1 (C_1_) was constituted based on phenotypic selection and intermating of the top 5% of 500 S_2_ families derived from each MYS population, test‐crossed and evaluated across moisture regimes. C_1_ was advanced to the next two cycles (C_2_ and C_3_) by intermating the top 5% selected individuals with high genomic estimated breeding values (GEBVs) for grain yield under drought and waterlogging stress. To estimate genetic gains, population bulks from each cycle were test‐crossed and evaluated across locations under different moisture regimes. Results indicated that the realised genetic gain under drought stress was 0.110 t ha^−1^ yr^−1^ and 0.135 t ha^−1^ yr^−1^, respectively, for MYS‐1 and MYS‐2. The gain was less under waterlogging stress, where MYS‐1 showed 0.038 t ha^−1^ yr^−1^ and MYS‐2 reached 0.113 t ha^−1^ yr^−1^. Genomic selection for drought and waterlogging tolerance resulted in no yield penalty under optimal moisture conditions. The genetic diversity of the two populations did not change significantly after two cycles of GS, suggesting that RC‐GS can be an effective breeding strategy to achieve high genetic gains without losing genetic diversity.

**Abbreviations**
ASI: anthesis‐silking interval
BLUP: best linear unbiased predictor
C_1_
: Cycle 1
C_2_
: Cycle 2
C_3_
: Cycle 3
CML: CIMMYT maize line
DT: drought tolerance
GEBV: genomic estimated breeding value
GS: genomic selection
GY: grain yield
MYS: multi‐parent yellow synthetic
RC‐GS: rapid‐cycle genomic selection
SNP: single‐nucleotide polymorphism
TC: test‐cross
WL: waterlogging tolerance.

## 1 INTRODUCTION

The rainfed system occupies a major part (∼80%) of maize mega‐environments in the Asian tropics, is largely dependent on prevailing weather conditions, and therefore extremely vulnerable to climate change effects (Prasanna, 2018). Drought and excess moisture stress are the two major abiotic stresses limiting maize production in large parts of the Asian tropics fed by monsoon rains. The per unit area productivity of the rainfed crop is usually less than half of maize in the irrigated system. The erratic and uneven distribution of monsoon rains occasionally causes drought or waterlogging at different crop growth stages within the same crop season and is the primary cause of the relatively low productivity of rainfed maize (Zaidi, Seetharam, & Vinayan, 2016a). Because farmers growing maize in such stress‐prone ecologies are not assured of returns, they are often hesitant to invest in recommended crop management, for example, adequate fertilizers, weed management, etc., which results in marginal agronomic conditions, and eventually further poor yields. Climate change effects are further threatening and challenging maize mega‐environments in the Asian tropics with high vulnerability and low adoption capacity (Cairns et al., 2012). Given the increasing climate variability, crop varieties need to be bred for resilience to variable weather conditions rather than tolerance to individual stresses in a specific situation or at a specific crop stage.

In the past, genotypes tolerant to individual stresses such as drought (Bänziger, Setimela, Hodson, & Vivek, 2006; Cairns & Prasanna, 2018; Edmeades, Bolanos, & Lafitte, 1992) and excess moisture (Ferreira, Coelho, Magalhaes, Gama, & Aluizio, 2007; Zaidi, Rafique, & Singh, 2007, 2003) have been successfully developed. However, the major challenge lies in putting together tolerance to multiple abiotic stresses and develop productive cultivars with combined stress tolerance. Previous studies have shown significant overlap between stresses such as drought and low nitrogen tolerance (Bänziger, Edmeades, & Lafitte, 2002; Zaidi, Rafique, Rai, Singh, & Srinivasan, 2004) and drought and waterlogging tolerance (Zaidi, Yadav, Singh, & Singh, 2008). Both drought and waterlogging tolerance are polygenic traits with significant additive effects conferred by many chromosomal regions. Crop improvement for tolerance to these stresses and building resilience for both in the same genetic background involves most chromosomal regions. Marker‐assisted recurrent selection (MARS), a method designed to increase the frequency of additively‐inherited favorable alleles in elite breeding populations, has been widely used in plant breeding where a few markers significantly associated with the phenotypic trait are employed (Bernardo, 2016, 2008). Genomic‐assisted breeding (or genomic selection) incorporates all available marker information simultaneously into a model to predict the genetic value of the candidate for selection (Meuwissen, Hayes, & Goddard, 2001) and population advancement through RC‐GS based on genetically estimated breeding values (GEBVs) without phenotyping in each cycle (Massman, Jung, & Bernardo, 2013). RC‐GS increases the genetic gain by reducing the length of the selection cycle, as has been exemplified in maize, where rapid cycling recombination was successfully used for improving biparental populations for drought tolerance (Beyene et al., 2015; Vivek et al., 2017) and multi‐parent populations for grain yield under optimal moisture conditions (Zhang et al., 2017). For RC‐GS within biparental populations, prediction accuracy is achieved (Crossa et al., 2014; Zhang et al., 2015). However, predictions across biparental populations will be poor if unrelated biparental populations with different allelic diversity are used as the training population (Zhang et al., 2017). The limited allelic diversity in one genetic background that occurs in biparental populations can be overcome by using multi‐parent populations with greater allelic diversity and from different genetic backgrounds (Verhoeven, Jannink, & McIntyre, 2006), along with increased polymorphism and recombination of biparental populations (Ahfock, Wood, Stephen, Cavanagh, & Huang, 2014).

**Core Ideas**

- Rapid cycle genomic selection (RC‐GS) to improve combined abiotic stress tolerance in tropical maize is relatively new.
- Genomic estimated breeding value (GEBV)‐based selection of superior phenotypes for targeted stresses leads to rapid genetic gains.
- Multi‐parent populations involving trait donors and elite lines to implement RC‐GS resulted in gains across stressed and non‐stressed environments.
- Populations may not be equally amenable, thus determining constituents in the base population and its constitution is a critical factor to GS.

Theoretical and simulation results show that genomic‐enabled prediction accuracy of multi‐parent populations is higher than the accuracy achieved within a single population (Hoffstetter, Cabrera, Huang, & Sneller, 2016; Lehermeier et al., 2014). RC‐GS was implemented in multi‐parent populations derived from 18 elite tropical maize inbred lines and improved for four selection cycles, including a first cycle based on phenotypic selection across locations under optimal growing conditions and three cycles based on GEBVs without phenotyping (Zhang et al., 2017). Results indicated that the realized gain in grain yield from C_1_ to C_4_ was 0.100 ton ha^−1^ yr^−1^. Integrated application of RC‐GS in a breeding pipeline provided a powerful combination to fast‐track the development of stress resilient maize for stress‐prone agroecologies by reducing the length of breeding cycles as well as saving on the cost of phenotyping in each cycle. The present study was initiated in the rainy season of 2011. Ten selected tropical maize inbred lines from each of the two major CIMMYT heterotic groups (HG) were intermated twice within HG to constitute two multi‐parent yellow synthetic (MYS) populations. The cycle 0 (C_0_) training set for the two populations was constituted by bulking equal numbers of seeds from each cob harvested after the second intermating. A total of 500 ear‐to‐row S_2_ families derived from C_0_ were genotyped with SNP markers using a genotyping‐by‐sequencing (GBS) platform. Their test crosses were phenotyped across locations in the Asian tropics under managed drought, managed waterlogging stress and optimal moisture conditions. One cycle of phenotypic selection (C_0_–C_1_) and two cycles of RC‐GS (C_1_–C_3_) were carried out, and test‐cross progenies from each selection cycle were evaluated across moisture regimes. The main objectives of this study were: (1) to assess the realized genetic gains under different moisture regimes after two cycles of rapid cycling of multi‐parent populations using GS, and (2) to investigate the changes in genetic diversity of the populations subjected to two cycles of RC‐GS.

## 2 MATERIALS AND METHODS

### 2.1 Constitution of training populations, cycle 0 (C_0_)

**FIGURE 1:**
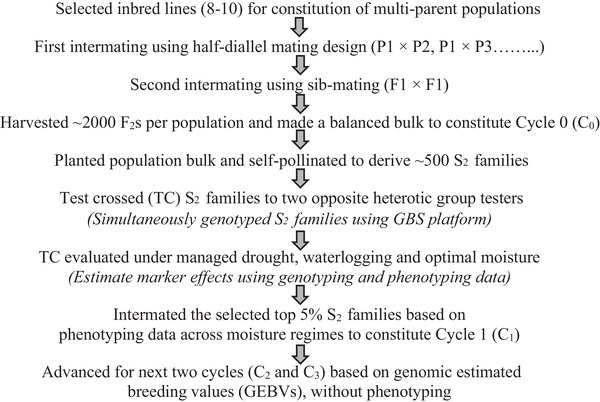
Steps in the breeding scheme used for the constitution of base populations and their advancement using rapid‐cycle genomic selection (RC‐GS)

The RC‐GS experiment was designed and implemented in 2011. The steps in the breeding scheme used for RC‐GS are shown in Figure 1. A total of 10 advanced stage tropical maize inbred lines from each heterotic group (HG‐A and HG‐B), including four each of drought and waterlogging tolerant lines and two elite high yielding lines (Table 1) were intermated twice. The drought trait donor lines were derived from the 9^th^ cycle of the CIMMYT drought tolerant yellow (DTY) populations, which were constituted during the mid‐1980s using 25 putative drought tolerant sources, including Tuxpeno Sequia C_8_, Latente, Michoacan 21, Suwan‐1, crosses of CIMMYT populations 22, 32, 62, 64 and 66, landraces, Corn Belt hybrids, and germplasm from Thailand, Brazil and South Africa (Edmeades & Deutsch, 1994). Waterlogging tolerant lines were derived from a waterlogging synthetic variety developed during 2005–2006 at the CIMMYT‐Asia Maize Program involving three lines from the population Suwan‐1, five CIMMYT maize lines (CMLs) and three CIMMYT Asia (CA) lines identified from line evaluation trials under managed waterlogging (Zaidi, Rafique, Singh, & Srinivasan, 2002). The first intermating was done using half‐diallel mating among selected inbred lines within each group. In the second intermating, an equal number of seeds was pooled from each harvested cob from a half‐diallel nursery of two populations to form a balanced bulk. Using seed from balanced bulk, 50 rows 5.0 m long were planted separately for each population. Intermating was done using bulk sibbing methods by dividing each population nursery into two equal halves (of 25 rows each) and pollinating the first half with bulk pollen collected from the second half and vice‐versa. After two rounds of intermating, cycle 0 (C_0_) of the two multi‐parent yellow synthetic (MYS) populations were formed by bulking an equal number of seed from the harvested cobs, and designated as MYS‐1 and MYS‐2.

**TABLE 1:** Inbred lines involved in the constitution of two heterotic multi‐parent, yellow synthetic (MYS) populations

Inbred lines	Heterotic group	Key traits
*Multi‐parent yellow synthetic population‐1 (MYS‐1)*		
CAL146	A	Drought tolerant
VL108871	A	Drought tolerant
VL108851	A	Drought tolerant
ZL11884	A	Drought tolerant
VL1010090	A	Waterlogging tolerant
CAL14101	A	Waterlogging tolerant
ZL11447	A	Waterlogging tolerant
VL1018820	A	Waterlogging tolerant
CIL12102	A	High‐yielding elite line
ZL11959	A	High‐yielding elite line
*Multi‐parent yellow synthetic population‐2 (MYS‐2)*		
CAL157	B	Drought tolerant
VL062623	B	Drought tolerant
VL109086	B	Drought tolerant
CAL1733	B	Drought and waterlogging tolerant
VL109138	B	Waterlogging tolerant
ZL182115	B	Waterlogging tolerant
ZL182116	B	Waterlogging tolerant
CAL1436	B	Waterlogging tolerant
ZL182117	B	High‐yielding elite line
CIL12180	B	High‐yielding elite line

### 2.2 Formation of cycle 1 (C_1_)

Each population was planted in 50 rows measuring 5.0 m and self‐pollinated to derive S_2_ families. The S_2_ families (about 510 from each population) were test‐crossed with opposite heterotic tester lines: CML 286 (HG‐A) and CML 451 (HG‐B). The test crosses (TCs) were evaluated across locations under managed drought and waterlogging stresses, and under optimal moisture conditions using standard phenotyping protocols (Zaidi, Vinayan, & Seetharam, 2016b; Zaman‐Allah et al., 2016). Data on key agronomic traits were recorded at each location, including plant height (PH), ear height (EH), root lodging (RL), stem lodging (SL), senescence (SN), anthesis date (AD), silking date (SD), ears per plant (EPP) and grain yield (GY) based on cob weight and moisture content (MOI) at harvest in all the trials. Data on brace roots (BR) were recorded only in waterlogging trial locations. Using across‐location analysis within each type of environment, the top‐ranking entries under optimal moisture and drought and waterlogging stresses were identified. This was followed by across‐environment selection, where the top 5% of the progenies across moisture regimes were identified (Table 2). Their female parents (S_2_ families) were planted using remnant seeds and intermated to constitute C_1_ for the two populations.

**TABLE 2:** Across‐location grain yield (t ha^−1^) of S_2_ family test crosses under optimal moisture (GY‐Opt), managed drought (GY‐DT) and waterlogging stress (GY‐WL), and performance of selected families relative to trial means in each environment

	Mean GY of the trial			Mean GY of selected fractions (top 5% families) and checks		
Entries	GY‐DT	GY‐WL	GY‐Opt	GY‐DT	GY‐WL	GY‐Opt
	t ha^−1^					
MYS‐1	3.11	2.65	6.37	4.11	3.33	7.11
MYS‐2	3.09	2.71	6.18	3.69	3.26	6.73
Checks				3.29	2.43	7.52

	Performance (% increase in GY) of selected fractions					
	Over trial mean			Over check mean		
	%					
MYS‐1	32.2	25.7	11.6	24.92	37.04	−5.45
MYS‐2	19.4	20.3	8.9	12.16	34.16	−10.51

### 2.3 Estimation of marker effects and genomic estimated breeding values

For each parental line, DNA was extracted by bulking equal amounts of leaf tissue from 20 individual plants. All the parents of both the populations were genotyped using 1256 SNPs at LGC Genomics, London. A total of 342 and 312 polymorphic markers for MYS‐1 and MYS‐2, respectively, were identified with maximum polymorphism information and used for genotyping the S_2_ families to estimate marker effects. Marker effects were estimated based on genotypic and phenotypic data for S_2_ families using the ridge regression best linear unbiased prediction (RR‐BLUP) method in R‐software (Crossa et al., 2010; Zhang et al., 2017). The Genomic Best Linear Unbiased Prediction (G‐BLUP) model in the BGLR package (VanRaden, 2008) was used to estimate GEBVs in C_1_ and C_2_ for advancement to the next cycle without phenotyping.

### 2.4 Formation of cycle 2 and cycle 3 using rapid cycling GS

Twenty rows measuring 5.0 m each were planted using bulk seeds of C_1_ of the two MYS populations, and each plant in the two populations was genotyped using 300 polymorphic SNP markers. The GEBV of each plant was estimated using GBLUP (genomic best linear unbiased prediction) using the BGLR package (VanRaden, 2008). The top 5% of individuals with high GEBVs for grain yield under drought and waterlogging stress in each population was identified, tagged and intermated using the bulk‐sibbing method. At harvest, an equal number of seed from the cob of each selected plant was bulked to constitute cycle 2 (C_2_). In the next season, 20 rows of C_2_ bulk seeds for each population were planted separately and each plant was genotyped using 300 polymorphic SNP markers for MYS‐1 and MYS‐2. GEBVs for each plant were estimated and the top 5% of progenies with high GEBVs for grain yield under stress were selected and intermated using the same procedure as above. After harvest, a balanced bulk was formed to constitute cycle 3 (C_3_).

### 2.5 Assessing genetic gains with selection cycles along with benchmark check hybrids

Seeds of all selection cycles (C_1_, C_2_ and C_3_) were increased and, in the next season, test‐crossed with heterotic tester lines. The test‐crosses were evaluated along with five check hybrids, including two internal checks (stress tolerant hybrids from CIMMYT‐Asia) and three commercial hybrids. Multi‐location evaluation trials were conducted across moisture regimes, including optimal moisture, managed drought and waterlogging at carefully selected locations (Table 3) where the desired level of stress was applied at the targeted crop growth stage at the desired level of intensity. All the field phenotyping trials were constituted using an alpha (0,1) lattice design with two replications using Field‐book software (Vivek, Kasango, Chisoro, & Magorokosho, 2007). Entries were planted in two‐row plots 4.0 m in length and spacing of 0.75 m by 0.2 m. Plots were over‐sown and later thinned to achieve a plant population of 66,666 plants ha^−1^. Optimal moisture trials were planted in a well‐drained field during the rainy season and supported with supplemental irrigation during the intermittent dry spell. The recommended package of practices for maize was followed at each location to keep the crop free from any nutrient deficiency/toxicity and biotic pressure (disease, insects or weeds). Managed drought stress trials were conducted during the dry season, when drought stress was imposed at the reproductive stage by managing the irrigation schedule (Zaman‐Allah et al., 2016). Managed waterlogging stress trials were conducted during the rainy season, when the stress treatment was applied by flooding the field at knee high level (at the V_5_‐V_6_ growth stage) and the water level was maintained stagnant at a depth of 10 ± 0.5 cm continuously for seven days by supplying water through need‐based supplemental irrigation at a rate that exceeded infiltration and evaporation. After the stress treatment, the field was completely drained and irrigation resumed as per crop needs (Zaidi et al., 2016b). Data were recorded on primary and secondary traits in the trials conducted under various moisture regimes using standard phenotyping protocols (Zaidi et al., 2016b; Zaman‐Allah et al., 2016).

**TABLE 3:** Phenotyping locations in different stress‐prone ago‐ecologies

Number	Trial code	Entries	Reps	Rows/Plot	Locations	GPS coordinates
*Managed drought stress*						
1	MYS‐TC‐Loc1	25	3	2	Godhara	22.77° N, 73.61° E
2	MYS‐TC‐Loc2	25	3	2	Aurangabad	19.76° N, 75.28° E
3	MYS‐TC‐Loc3	25	3	2	Hyderabad	17.51° N, 78.27° E
*Managed waterlogging stress*						
1	MYS‐TC‐Loc1	25	2	2	Begusarai	25.4° N, 86.12° E
2	MYS‐TC‐Loc2	25	2	2	Varanasi	25.26° N, 82.99° E
*Optimal moisture*						
1	MYS‐TC‐Loc1	25	2	2	Daulatabad	17.71° N, 78.20° E
2	MYS‐TC‐Loc2	25	2	2	Ludhiana	30.54° N, 75.50° E
3	MYS‐TC‐Loc3	25	2	2	Hyderabad	17.51° N, 78.27° E

### 2.6 Statistical analysis of field trial data

Phenotypic data were collected in all the trials and locations for agronomic traits, including PH, EH, RL, SL, SN, AD, SD, EPP, GY and MOI at harvest, whereas BR was recorded only in waterlogging trials. Phenotypic data for each site were analyzed using the residual maximum likelihood (REML) procedure in Field‐book (Vivek et al., 2007). Grain yield was estimated by adjusting grain moisture content to 12.5%. Best linear unbiased estimators (BLUEs) were calculated for each entry within each site. Across‐site analysis was also done using Field‐Book software, where genotype was treated as random and site as a fixed effect. Regression analysis of selection cycle means for grain yield over selection cycles (C_1_, C_2_ and C_3_) was used to assess the genetic gain responses with RC‐GS.

### 2.7 Genetic diversity analysis

Based on genomic data, we computed two genetic diversity indices between the families of the different selection cycles as well as the parents (Zhang et al., 2017). Shannon's Diversity Index was calculated for each selection cycle as

$$\frac{1}{A}\Sigma_{a\;=\;1}^{A}{\hat{P}}_{a}\mathrm{In}\left({\hat{P}}_{a}\right)$$

where ${\hat{P}}_{a}$ is the frequency of the major allele in the a^th^ marker over the entire sample, and A is the total number of markers. The expected proportion of heterozygous loci per individual was computed as the mean of heterozygosity for each marker as

$$0\leq \frac{1}{L}\Sigma_{l\;=\;1}^{L}\;\left(1-\Sigma_{a\;=\;1}^{n_{i}}{\hat{P}}_{\mathrm{la}}^{2}\right)\leq 1$$

where ${\hat{P}}_{\mathrm{la}}$ is the frequency of the major allele in the a^th^ marker of the l^th^ individual, and L is the number of individuals. A graphic representation of genetic diversity was achieved by measuring the distance between genotypes by the DMATCH distance in PROC DISTANCE capability of SAS software. The distance matrix was dimensionally reduced using multidimensional scaling (PROC MDS capability in SAS software) and the two first dimensions were plotted (SAS/GRAPH capability) to compare cycle genome composition (SAS Institute Inc., 2017).

The diversity between the three cycles of selection and the parents (groups in Table 6) and within them was analyzed by molecular analysis of variance. We selected this method instead of other available methods because it does not assume Mendelian gene frequencies. Analyses were performed in R package using the capabilities of the library *poppr* (R Core Team, 2019). Variance components were estimated using ade4, and 1000 permutations were done in order to estimate significance values.

## 3 RESULTS

### 3.1 Heritability of GY and response of selected and non‐selected traits

Test‐cross progenies from three selection cycles (C_1_, C_2_ and C_3_) of two populations (MYS‐1 and MYS‐2) along with five check hybrids including two of CIMMYT's stress resilient hybrids as internal genetic gain checks and three popular commercial hybrids in rainfed ecologies were used for evaluation across locations under different moisture regimes (Table 3). The heritability of GY under managed drought stress ranged from 0.58 at Godhara to 0.96 at Aurangabad (Table 4). The heritability of GY was relatively low under waterlogging, ranging from 0.53 at Begusarai to 0.84 at Varanasi, but was relatively high under optimal moisture, ranging from 0.63 at Ludhiana to 0.82 at Hyderabad. Data from locations with poor heritability (< 0.40 for drought or waterlogging and < 0.50 for optimal moisture trials) were rejected and not used in further analyses.

**TABLE 4:** Descriptive statistics of grain yield (t ha^−1^) of MYS test crosses evaluated across‐location under managed drought, waterlogging and optimal moisture

	Drought			Waterlogging		Optimal moisture		
	GOD^a^	AUR	HYD	BGS	VAR	DLB	LUD	HYD
Mean	2.69	2.61	2.60	1.77	1.93	6.66	6.52	6.68
Min	1.96	2.02	2.63	0.51	0.47	5.16	4.21	4.42
Max	3.13	3.01	3.66	2.19	2.82	7.4	8.07	8.19
MSe	0.42	0.47	0.32	0.26	0.55	0.62	1.83	0.51
*p‐sig*	^**^	^***^	^***^	^**^	^***^	^*^	^**^	^**^
*H^2^*	0.58	0.96	0.82	0.53	0.84	0.71	0.82	0.63

^a^GOD = Godhara, AUR = Aurangabad, HYD = Hyderabad, BGS = Begusarai, VAR = Varanasi, DLB = Daulatabad and LUD = Ludhiana; *, **, and *** indicate statistical significance at .05, .01, and .001, respectively.

There was no significant change in PH, AD, ASI or EPP from C_1_ to C_3_. However, PH increased significantly in both the populations in C_3_ under optimal moisture conditions (Table 5). In general, there was significant genotypic variability among test entries for all the traits, except AD and ASI under optimal moisture (Table 5). Days to anthesis were maintained at 64.9 ± 0.67 and 66.2 ± 0.74 in MYS‐1 and MYS‐2, respectively, under optimal moisture after two cycles of selection with good synchrony between male and female flowering (< 3.0 days). A similar trend of non‐significant change in days to anthesis was maintained under drought and waterlogging stresses as well. However, ASI fell significantly from C_1_ to C_3_ in MYS‐1 under both drought and waterlogging, and under drought in the case of MYS‐2. There was no significant change in EPP under optimal moisture; however, it increased significantly in C_3_ in comparison to C_1_ in both populations under drought as well as waterlogging stress.

**TABLE 5:** Across location performance of the test‐cross progenies of MYS populations under managed drought, waterlogging and optimal moisture conditions

	Drought stress					Waterlogging stress					Optimal moisture				
	PH	AD	ASI	EPP	GY	PH	AD	ASI	EPP	GY	PH	AD	ASI	EPP	GY
Entries^a^	cm	d	d	No.	t/ha	cm	d	d	No.	t/ha	cm	d	d	No.	t/ha
MYS1‐C1‐TC	200.5b,c,d,e,f	79.9	5.2a	0.8d,e	2.58c,d	108.8c,d	56.8e	4.3a	0.8b,c,d,e	2.11b,c,d	167.7e	64.0	2.1	0.9b	5.80d
MYS1‐C2‐TC	193.7c,d,e,f	80.6	3.2b,c	0.8e	2.78b,c	112.7c,d	57.2d,e	2.4b,c	0.9a,b,c,d	2.33a,b	179.8c,d	65.6	1.7	1.0a,b	5.96d
MYS1‐C3‐TC	192.1c,d,e,f	79.6	3.8a,b,c	0.9b,c	3.05a,b	112.5c,d	58.5c,d,e	2.3b,c	1.0a	2.25a,b,c	172.9d,e	65.2	2.8	0.9b	5.86d
MYS2‐C1‐TC	184.0f	84.3	3.8a,b,c	0.9b,c,d,e	2.76b,c	116.0c,d	58.9c,d	2.6b,c	0.7e,f	2.01,d	179.7c,d	66.6	2.0	0.9b	6.23c,d
MYS2‐C2‐TC	185.8e,f	83.6	2.2c	0.9c,d,e	2.89b,c	107.8c,d	59.4b,c	4.5a	0.8a,b,c,d,e	2.04c,d	185.6c	65.2	2.6	0.9b	6.44c,d
MYS2‐C3‐TC	188.7 d,e,f	83.2	2.0c	1.0b	3.30a	112.5c,d	60.0b,c	3.0b	0.9a,b	2.45a	190.4b,c	66.9	2.1	0.9b	6.33c,d
Internal Check‐1 (CAH‐1511)	205.0 a,b,c,d,e	81.8	3.7a,b,c	0.9b,c	2.24e	132.4a,b	58.4c,d,e	1.6c,d,e	1.0a,b	1.92d	210.6a	66.2	2.0	1.0a,b	8.15a,
Internal Check‐2 (CAH‐153)	206.4 a,b,c,d	82.6	4.2a,b,	1.2a	2.6c,d	123.2b,c	60.1b,c	2.2b,c,d	0.8c,d,e	1.83d	197.2b	64.5	2.0	1.0a,b	7.02b,c
Commercial Check‐1 (DKC9108)	211.5 a,b,c	80.1	3.3b,c,	1.0b,c	2.22e	112.6c,d	63.1a	1.0e	0.6f	1.21e	183.1c,d	66.2	2.1	1.0a,b	7.00b,c
Commercial Check‐2 (PAC745)	218.2a,b	84.6	3.6a,b,c	0.9b,c,d	2.24e	119.8c,d	59.2c,d	1.9c,d,e	0.9a,b,c	0.95f	188.7b,c	66.4	1.8	1.0a,b	6.52b,c,d
Commercial Check‐3 (HTMH5101)	223.0a	79.4	2.9b,c	0.9c,d,e	2.29d,e	137.0a	61.0b	1.2d,e	0.8d,e,f	1.25e	197.7b	64.0	1.8	1.1a	7.43 a,b
Mean	200.80	81.79	3.45	0.93	2.63	117.74	59.33	2.45	0.84	1.86	186.67	65.53	2.09	0.96	6.61
LSD (0.05)	19.82	2.45	1.22	0.10	0.33	12.11	2.08	1.05	0.14	0.27	11.2	ns	ns	0.19	0.95
MSe	291.51	7.83	2.41	0.01	0.39	68.82	2.44	3.05	0.02	0.28	189.0	1.2	0.6	0.01	0.99
Min	184.04	79.40	2.20	0.82	2.22	107.78	56.80	1.00	0.64	0.84	167.7	64.00	1.70	0.89	5.80
Max	223.04	84.6	5.20	1.16	3.30	136.96	63.10	4.50	0.97	2.45	210.6	66.90	2.80	1.06	8.15

^a^PH = Plant height, AD = Anthesis date, ASI = Anthesis‐silking interval, EPP = Ears per plant and GY = grain yield; MYS = Multiparent yellow synthetic, C1, C2 and C3 = Cycle 1, Cycle 2, and Cycle 3, respectively; Entries followed by a common letter are not different based on LSD (0.05)

**TABLE 6:** Analysis of molecular variance (AMOVA) among three cycles of selection and parents (groups) in two multiparent yellow synthetic (MYS) populations

**MYS‐1**	**Degree of Freedom**	**Sum Square**	**Mean Square**	**Sigma**	**%**	**Phi**	**Alternative hypothesis**	**P‐value**
Between groups^a^	3	1668	555.9	0.84	1.78	0.0178	greater	0.00100
Between samples within groups	1390	60261	43.35	0	0	0	greater	1.0000
Within samples	1394	64551	46.31	46.3	98.2	0.0178	less	0.9980
Total	2787	126480	45.38	47.1				

**MYS‐2**	**Degree of Freedom**	**Sum Square**	**Mean Square**	**Sigma**	**%**	**Phi**	**Alternative hypothesis**	**P‐value**
Between groups^a^	3	2067	689.1	1.01	2.14	0.0214	greater	0.0010
Between samples within groups	1436	62723	43.68	0	0	0	greater	1.0000
Within samples	1440	66800	46.39	46.4	97.9	0.0214	less	0.9790
Total	2879	131590	45.71	47.4				

a groups represent three selection cycles (C_1_, C_2_, and C_3_) and parents.

Of the two stresses, the effect of waterlogging was relatively much more severe compared to drought, which was reflected in the lowest mean yield of the trial under waterlogging. The advanced GS cycle showed significant improvement in grain yield under both drought and waterlogging stress in comparison to commercial checks as well as internal genetic gain checks. In general, the performance of GS cycle TCs was better than the performance of both internal and commercial hybrid checks, under drought as well as under waterlogging stress. Though under optimal moisture, two check hybrids (internal check‐1 and commercial check‐3) were significantly superior to all test entries, other check hybrids were on a par with GS cycle test crosses. Rapid cycling recombination of GS for combined drought and waterlogging tolerance did not affect the performance of both the populations under optimal moisture, as there was no significant change in grain yield under optimal moisture with two cycles of selection.

### 3.2 Realized genetic gains from recombination of GS for grain yield

An assessment of relative grain yields of population test crosses derived from different GS recombination cycles showed varied responses under different moisture regimes (Table 4). Under drought stress, MYS‐1 showed an 8.4% gain in grain yield per cycle, whereas MYS‐2 had a 9.7% gain per cycle. The gain was almost linear from C_1_ to C_3;_ however, both populations showed relatively more gain between C_2_ to C_3_ (9.0 and 14.1%) in comparison to C_1_ to C_2_ (7.2 and 4.4%), respectively. In the case of waterlogging stress, the genetic gain per GS cycle was low with MYS‐1 (3.6%), whereas MYS‐2 showed relatively high gains (11.3%). MYS‐1 responded relatively better from C_1_ to C_2_ with a gain of 10.95%, but from C_2_ to C_3_ there was a slight yield loss (−3.4%). MYS‐2 responded differently under waterlogging with a relatively smaller gain (1.5%) from C_1_ to C_2_ and high gain (20.7%) from C_2_ to C_3_. Assessment of mean grain yields of population TCs for different GS cycles under optimal moisture showed that in both populations, the gains from C_1_ to C_3_ were nominal (0.55 and 0.74% for MYS‐1 and MYS‐2, respectively). Rapid cycling recombination of GS in C_2_ and C_3_ was based on simultaneous selection for drought and waterlogging tolerance; however, yields in advanced cycles of both populations were maintained under optimal moisture after two cycles of GS‐based recombination. While comparing the performance of GS cycles test‐crosses with commercial checks, the yield in C_1_ (constituted based on phenotypic selection) was on a par or slightly better than that of the best check hybrid, and was further improved with rapid cycling recombination of GS, as the mean grain yield in advanced cycles was significantly higher than that of all the checks used in the trials under managed drought or waterlogging stresses. Under optimal moisture there was not much change with GS, as the checks were superior to all the GS cycle test crosses of both populations.

**FIGURE 2:**
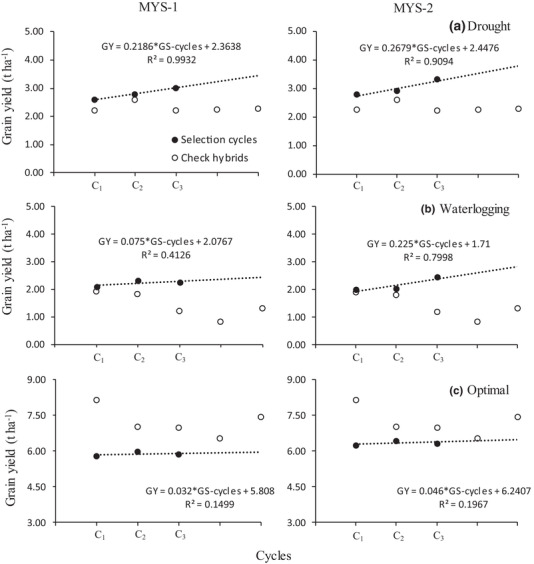
Regression of mean grain yield of selection cycles over number of selection cycles (C_1_, C_2_ and C_3_) (indicating genetic gains under (a) drought stress, (b) waterlogging stress and (c) optimal moisture conditions with genomic selection in two multiparent populations (MYS‐1 and MYS‐2). Open dots indicate the performance of check hybrids evaluated along with selection cycles under different moisture regimes

Regression analysis of mean grain yields over number of selection cycles across locations in different cycles showed that under drought stress, both populations responded alike to rapid cycle recombination of GS and the per cycle gain from C_1_ to C_3_ was largest under drought stress, i.e., 219 kg cycle^−1^ and 268 kg cycle^−1^ in MYS‐1 and MYS‐2, respectively (Figure 2). Under waterlogging stress, MYS‐1 did not show significant improvement, as per cycle gain was only 75 kg cycle^−1^ due to yield loss between C_2_ and C_3._ However, MYS‐2 showed significant gain with RC‐GS at 225 kg gain cycle^−1^. Under optimal moisture, both populations showed no significant gain or loss due to combined GS for the two stresses.

### 3.3 Heterozygosity and genetic diversity of the rapid cycle recombination of GS

**FIGURE 3:**
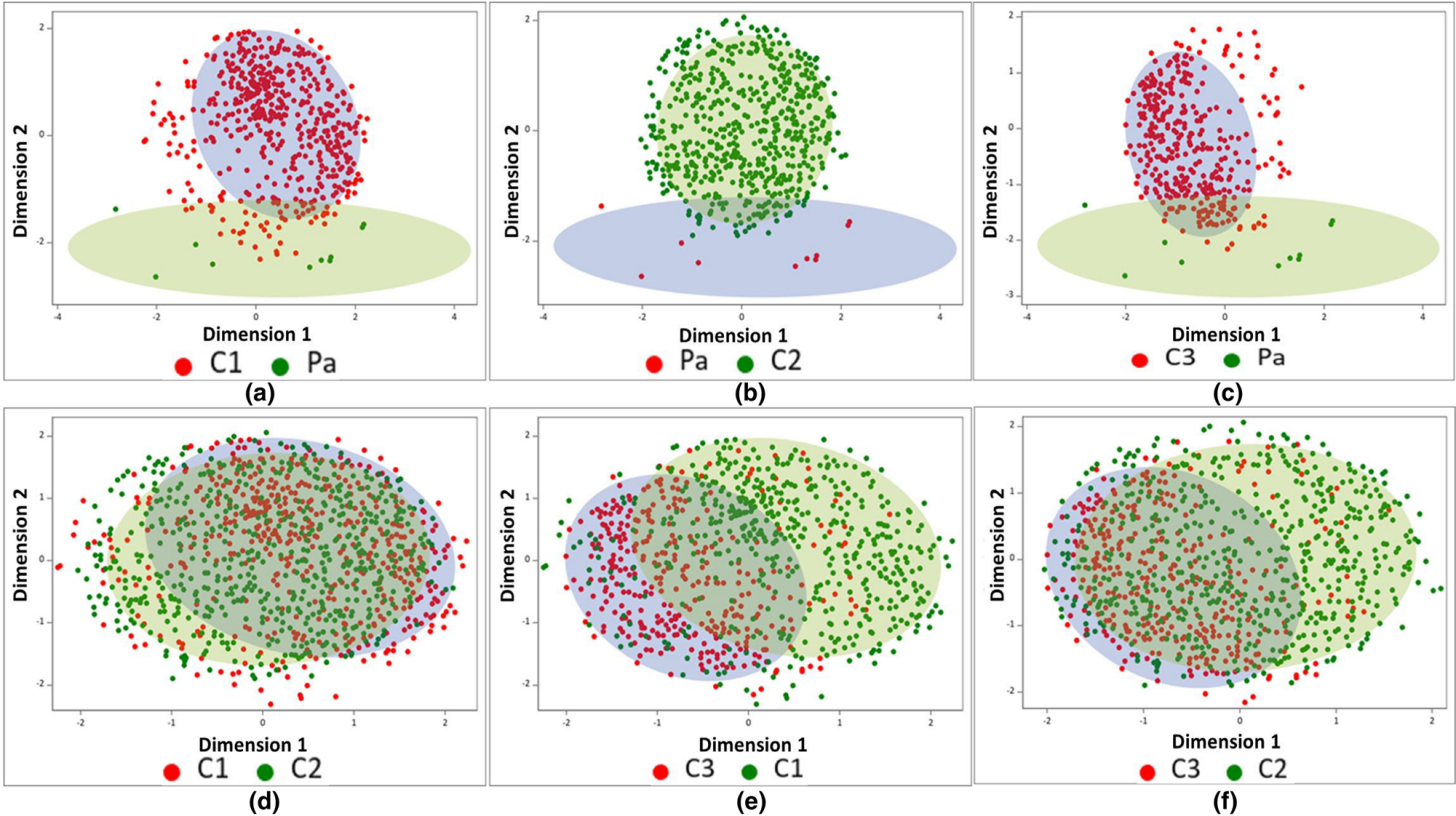
Changes in population structure with selections in MYS‐1 (Pa = parents, C1, C2 and C3 = cycle‐1, 2 and 3, respectively). Pairwise Phi statistics: Pa‐C1: 0, Pa‐C2: 0, Pa‐C3: 0.019, C1‐C2: 0.009, C1‐C3: 0.034, and C2‐C3: 0.019

**FIGURE 4:**
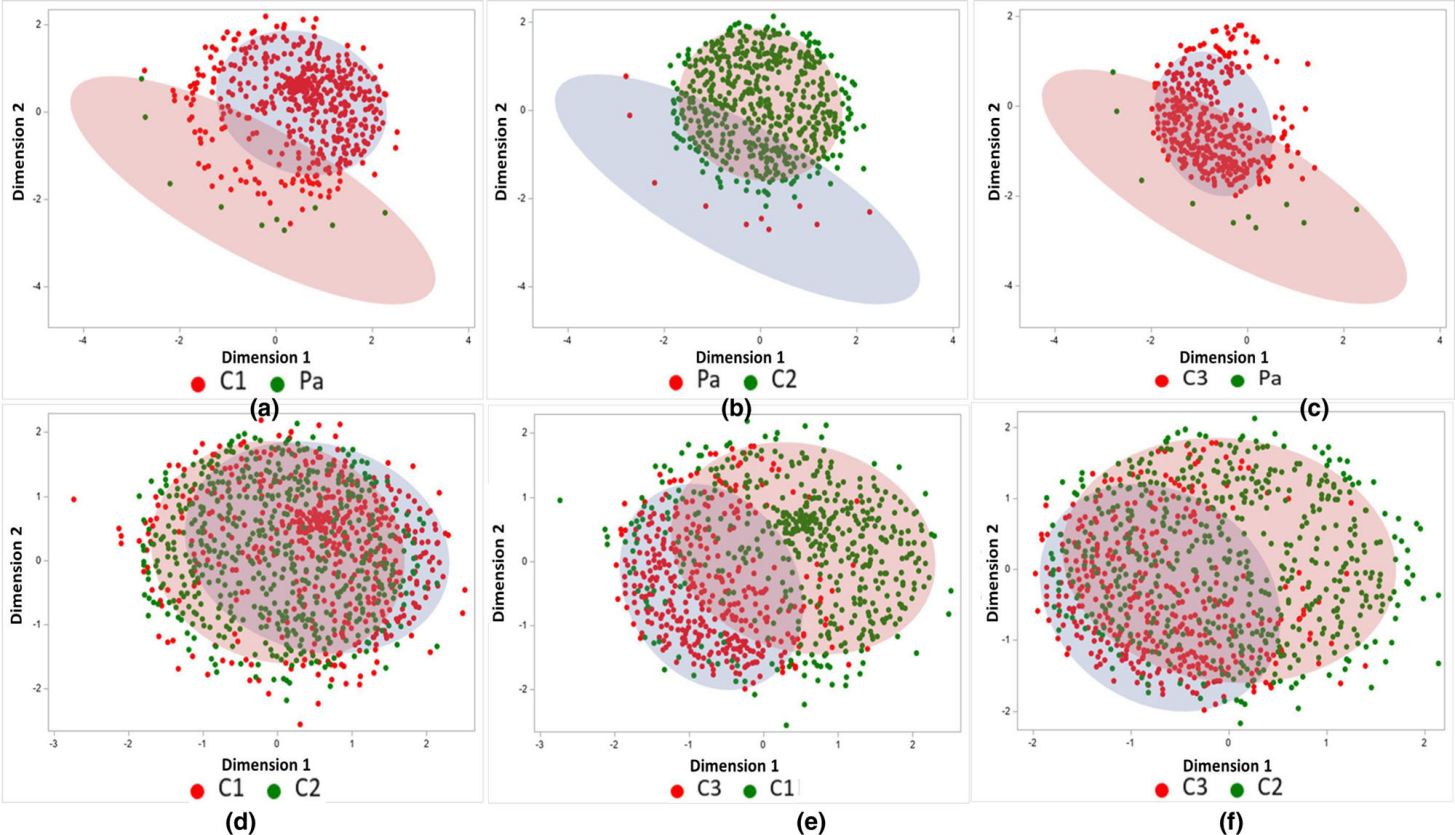
Changes in population structure with selections in MYS‐2. (Pa = parents, C1, C2 and C3 = cycle‐1, 2 and 3, respectively). Pairwise Phi statistics: Pa‐C1: 0, Pa‐C2: 0.003, Pa‐C3: 0.034, C1‐C2: 0.011, C1‐C3: 0.043, and C2‐C3: 0.017

The genetic diversity structure of 10 parents involved in the constitution of each population along with three selection cycles are presented in Figures 3a–c and Figure 4a–c. The member of each cycle was well‐spread along the two dimensions. The spread of parents along the two dimensions shows that parents involved in MYS‐2 were relatively more diverse than those involved in MYS‐1. The dispersal along the two axes clearly shows that new progenies were largely new recombinants compared to the parents (Figure 3a). A few C_1_ families are located between dimensions 1 and 2, close to the original parents located in this region in Figure 3a. The 10 parents of MYS‐1 along with the C_2_ families are shown in Figure 3b. The C_2_ families and the parents are located between dimensions 1 and 2, clearly heading in the direction of two different axes, showing comparatively less similarity than the C_1_ progenies. Figure 3c, which includes the 10 parents of MYS‐1 and the C_3_ families, shows that C_3_ families and the parents are concentrated around their own dimension, revealing that C_3_ is more diverse than C_1_ and C_2_. However, a comparison of different populations (C_1_–C_3_) may be confounded by variation in population size and level of inbreeding in the different selection cycles (Figure 3d–f). Figure 3d and 3f represent the immediate cycles of C_1_, C_2_, and of C_2_, C_3_, respectively. Immediate cycles are intermingled in the two dimensions showing there is relatively low diversity in the immediate cycles, whereas in Figure 3e, C_1_ and C_3_ are separated in well‐defined dimensions, showing that they are more diverse compared to immediate cycles. A similar analysis of the MYS‐2 population is presented in Figure 4a–f. The spread of parents along the two dimensions, especially dimension 1, shows that parents involved in MYS‐2 were relatively more diverse than those in MYS‐1. The dispersal of progenies along two axes clearly shows that progenies are new recombinants largely diverse from the parents. Rapid cycle recombinants of GS added further diversity in C_2_ as well as in C_3_ (Figure 4b,c). A comparison of the progenies dispersal along two axes in three cycles shows similar trends as in MYS‐1, where immediate cycles (Figure 4d,f) were relatively less diverse, while diversity was relative more visible between C_1_ and C_3_ (Figure 4e).

**FIGURE 5:**
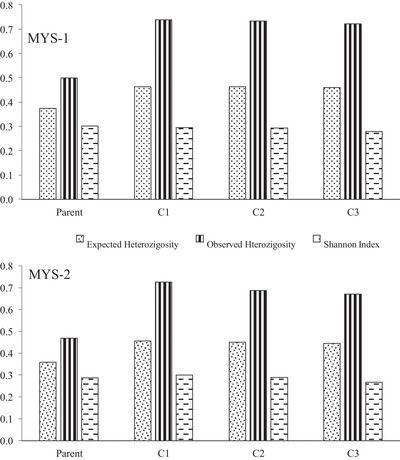
Expected and observed heterozygosity and Shannon's Diversity Index in parents, C_1_, C_2_ and C_3_ of the two multi‐parent yellow synthetics (MYS) populations

Shannon's Diversity Index and expected and observed heterozygosity of the 10 founders of MYS‐1, MYS‐2, C_1_, C_2_ and C_3_ are presented in Figure 5. There was a significant increase in diversity values in C_1_ constituents in comparison to the parents, which indicates the formation of new recombinants with two rounds of intermating. Trends in Shannon's Diversity Index and heterozygosity show that diversity did not decline in the next advanced cycles (C_2_) with rapid cycling recombination of GS. However, there was a nominal decrease in diversity in C_3_. Diversity analysis using Shannon's Diversity Index indicated that diversity was maintained even after two cycles of GS (Figure 5). Shannon's Diversity Index of C_3_ for both populations was more than 0.26, above the threshold value of 0.20 which is considered good diversity. The results clearly show that despite the significant genetic gains with genomic selection, the genetic diversity of the advanced population was maintained.

The molecular analysis of variance confirmed the differentiation between cycles and parental populations (Table 6). Even though the differentiation between populations explains only 1.78% (p‐value = .001) and 2.14% (p‐value = .001) for MYS1 and MYS2, respectively, there is a significant effect.

## 4 DISCUSSION

The expected gain from GS per unit time is defined as Δ*_G_*  =  *irσ*_A_/*y*, where *i* is the selection intensity, *r* is the selection accuracy, *σ*_A_ is the square root of the additive genetic variance, and *y* is the time needed to complete one selection cycle (Falconer & Mackay, 1996). Assuming equal selection intensities and genetic variance for both GS and phenotypic selection, greater gain per unit time is expected by reducing the duration of the selection cycle using GS.

In this study, gains were not consistent between cycles and under different selection environments. Under drought stress (Figure 2a), MYS‐1 showed linear gains from C_1_ to C_3,_ but in the case of MYS‐2, the gain was relatively higher between C_2_ and C_3_ (14.4%) than between C_1_ and C_2_ (4.4%). The regression analysis showed that the gain per cycle was higher in MYS‐2 (268 kg ha^−1^ cycle^−1^) compared to MYS‐1 (219 kg ha^−1^ cycle^−1^). The reason there were relatively more gains between C_2_ and C_3_ in both the populations may be because C_2_ was constituted based on one round of selection of recombinants in C_1_, and selection of new recombinants in C_2_ for constituting C_3_ might have increased desirable allele frequency for drought tolerance. In the case of waterlogging stress (Figure 2b), the trends in gains in two populations were relatively less consistent. MYS‐1 showed gain from C_1_ to C_2_ (10.95%) but there was nominal loss between C_2_ and C_3_ (‐3.4%), whereas in MYS‐2 there was nominal gain (1.5%) between C_1_ and C_2_ but C_2_ to C_3_ showed significant gain (20.7%). The trend was eventually reflected in the final gain across cycles, as MYS‐2 showed 225 kg ha^−1^ cycle^−1^, whereas MYS‐1 had only 75 kg ha^−1^ cycle^−1^. These findings suggest that response to GS may not be similar in all populations and may vary with the strength of the trait donors involved in constituting the base germplasm, as GS was based on selection of relatively superior individuals within populations when constituting advanced cycles. Schopp, Müller, Technow, and Melchinger (2017) carried out a simulation study by generating synthetic populations with 2 and 32 parents and found that sampling a few parents (2‐8) generates substantial sample linkage disequilibrium (LD) that carried over into synthetics through co‐segregation of alleles at linked loci, contributing towards higher prediction accuracy. When a larger number of parents are used in developing synthetics, it is the ancestral LD (between QTL and markers in the ancestral population of founders) between the parents that contribute to prediction accuracy, and hence may be much lower if the parental lines are highly unrelated. Inconsistency in genetic gains was also observed by Zhang et al. (2017) under optimal moisture conditions where a multi‐parent population was subjected to RC‐GS. Relatively greater gains under drought stress can be explained by the fact that CIMMYT's drought program is comparatively much stronger, and the donor lines involved in the constitution of the base population were derived from nine cycles of recurrent selection for drought tolerance (Edmeades & Deutsch, 1994).

Comparison of advanced cycle (C_3_) test‐cross performance with the best check hybrid (CAH‐153, one of the most stress resilient hybrids from the CIMMYT‐Asia program) showed that gains were relatively higher under waterlogging stress (25.3 and 37.3%) compared to drought stress (18.2 and 29.2%) in MYS‐1 and MYS‐2, respectively. It was also observed that even the performance in C_1_ of both populations under drought as well as waterlogging was on a par or better than that of the best check entry (Figure 2a,b). This suggests that careful constitution of the base population involving promising trait donors and phenotypic selection based on superior test‐cross progenies to constitute C_1_ helped to bring together desirable alleles for drought and waterlogging tolerance, and two cycles of GS followed by intermating further helped in the accumulation of desirable recombinants and increased the genetic gains across moisture regimes. The performance of different GS cycles under optimal moisture conditions (no targeted GS selection environment) indicated that there were nominal positive gains, 32 kg ha^−1^ cycle^−1^ in MYS‐1 and 46 kg ha^−1^ cycle^−1^ in MYS‐2. This may be explained by the fact that apart from the stress tolerant trait donors, elite high yielding lines were also involved in constituting the base populations, and the constitution of the first cycle (C_1_) was based on selecting superior test‐cross progenies across moisture regimes, including drought, waterlogging and optimal moisture. These findings suggest that abiotic stress tolerance may not necessarily be associated with yield penalties under optimal conditions, provided performance under optimal conditions is taken into account while constituting the base population and doing phenotypic selection for constituting C_1_. Using a trait‐based selection approach, Zaidi et al. (2008) identified maize inbred lines tolerant to both drought and waterlogging stresses and suggested that constitutive changes with selection and improvement for stress tolerance may result in improved performance of genotypes under both drought and excess moisture stresses, without any yield penalty under optimal moisture. A similar finding was reported in earlier studies on abiotic stress tolerance breeding using a conventional breeding approach (Bänziger et al., 2006).

In this study, we implemented two cycles per year, and it took eight crop seasons (4 years) from the first intermating of selected lines to constitute the base populations and harvest C_3_ seeds. Computing the yield gains (t ha^−1^ yr^−1^_)_ with GS from C_1_ to C_3_ showed that MYS‐1 gained 0.110, 0.038 and 0.015 t, while MYS‐2 gained 0.135, 0.113 and 0.025 t under drought, waterlogging and optimal moisture conditions, respectively. Beyene et al. (2015) reported a gain of 70 kg ha^−1^ yr^−1^ with RC‐GS for drought stress tolerance in biparental populations. Our study showed higher gains under drought stress which may be due to difference in base population structure, as we used multi‐parent populations, whereas Beyene et al. (2015) implemented RC‐GS using biparental populations. These findings suggest that a multi‐parent population may be a better choice for GS‐based breeding for polygenic traits such as drought and waterlogging tolerance, where more than one mechanism may confer stress tolerance in different situations, and especially for combining such traits. Zhang et al. (2017) reported a gain of 100 kg ha^−1^ yr^−1^ with RC‐GS for grain yield under optimal moisture using multi‐parent populations, which may not be comparable to the gains in our study, as we targeted GS for drought and waterlogging stress tolerance, while the main trait for GS was grain yield under optimal moisture in their study. However, there were slight yield gains under optimal moisture as well as significant gains for traits targeted for GS, that is, grain yield under drought and waterlogging stress. In this study, genetic gains per year for polygenic traits such as tolerance to drought and waterlogging stresses without any penalty under optimal moisture, clearly demonstrate the strength of GS‐assisted recombination. In terms of time efficiency, two cycles per year were completed in GS‐based selection, which is more efficient than conventional recurrent selection and cycle advancement where at least three seasons (1.5 years under our conditions) are needed per selection cycle, including deriving progenies, making test crosses, phenotyping test crosses, and conducting selection and recombination.

Based on simulation studies, Jannink, Lorenz, and Iwata (2010) cautioned about the possible decline in genetic variance due to RC‐GS. Genomic selection can also result in significantly less genetic variance over time largely because it reduces breeding cycle duration. In short‐term selection programs, rapid loss of genetic variance may not be an issue, while long‐term GS programs experiencing swifter losses of genetic variance could reduce the rate of genetic gain and eventually affect the efficiency of the breeding program. Genetic gains in GS for stem rust in wheat were reported by Rutkoski et al. (2015), who also found significant increases in inbreeding after one and two cycles of GS compared to C_0,_ greater than the expected value under random genetic drift for all populations. In our study, genetic diversity measured in terms of Shannon's Diversity Index and heterozygosity showed a significant increase in C_1_ compared to the parents, which may be explained by the formation of new recombinants with two rounds of intermating. With further advancement of the populations, there was only a nominal decline in their genetic diversity after two cycles of GS (Figure 5), which is in agreement with findings by Zhang et al. (2017), i.e., that there was no significant change in genetic diversity in the initial two cycles of GS, and a decrease only during the last GS cycles (C_3_ and C_4_).

## 5 CONCLUSIONS

Findings of this study are the first of its kind that have reported RC‐GS for simultaneous improvement of drought and waterlogging tolerance using multi‐parent synthetic populations. Realized genetic gains after two cycles of rapid cycle recombination of GS were 0.110 and 0.135 t ha^−1^ yr^−1^ under drought, and 0.038 and 0.113 t ha^−1^ yr^−1^ under waterlogging in MYS‐1 and MYS‐2 populations, respectively. Gains were relatively higher for drought stress than for waterlogging tolerance, and of the two populations, MYS‐2 responded comparatively better to RC‐GS for both stresses. The differential response of the two populations to RC‐GS suggested that the strength of lines/trait donors involved in constituting the base population plays a key role in the genetic gain with GS. Therefore, it would be more efficient to evaluate the population per se at the initial stage and move forward with selected potential population(s) for rapid cycling using GS to save on genotyping costs. Simultaneous GS for improved tolerance to two key abiotic stresses did not show any yield penalty under optimal moisture. The genetic diversity analysis of the parents and three cycles indicated that it increased from parents to the first constitution of the first cycle (C_1_) and narrowed down only slightly with the next two cycles of rapid cycling.

## CONFLICT OF INTEREST

The authors declare that there is no conflict of interest.

## Supporting information

Supplementary Table 1: Marker statistics of the markers used for genomic selection in MYS‐1
Supplementary table 2: Marker statistics of the markers used for genomic selection in MYS‐2
**Supplementary table 3**: MYS‐1 S2 families TC data from managed drought trials
**Supplementary table 4**: MYS‐1 S2 families TC data from managed waterlogging trials
**Supplementary table 5**: MYS‐1 S2 families TC data from optimal moisture trials
**Supplementary table 6**: MYS‐2 S2 families TC data from managed drought trials
**Supplementary table 7**: MYS‐2 S2 families TC data from managed waterlogging trials
**Supplementary table 8**: MYS‐2 S2 families TC data from optimal moisture trials
**Supplementary table 9**: Selection cycles TC trials data from managed drought trials
**Supplementary table 10**: Selection cycles TC trials data from managed waterlogging trials
**Supplementary table 11**: Selection cycles TC trials data from optimal moisture trials
